# *Herpes Simplex* Virus Type 1 Infects Enteric Neurons and Triggers Gut Dysfunction via Macrophage Recruitment

**DOI:** 10.3389/fcimb.2018.00074

**Published:** 2018-03-15

**Authors:** Paola Brun, Marsela Qesari, Peggy C. Marconi, Andromachi Kotsafti, Andrea Porzionato, Veronica Macchi, Reto A. Schwendener, Marco Scarpa, Maria C. Giron, Giorgio Palù, Arianna Calistri, Ignazio Castagliuolo

**Affiliations:** ^1^Department of Molecular Medicine, University of Padova, Padova, Italy; ^2^Department of Pharmaceutical and Pharmacological Sciences, University of Padova, Padova, Italy; ^3^Department of Life Sciences and Biotechnology, University of Ferrara, Ferrara, Italy; ^4^Esophageal and Digestive Tract Surgery Unit, Veneto Institute of Oncology IOV–IRCCS, Padova, Italy; ^5^Department of Neurosciences, University of Padova, Padova, Italy; ^6^Institute of Molecular Cancer Research, University of Zurich, Zurich, Switzerland

**Keywords:** neurotropic virus, enteric neuropathies, inflammation, neuromuscular dysfunction, macrophage recruitment

## Abstract

*Herpes Simplex* Virus type 1 (HSV-1), a neurotropic pathogen widespread in human population, infects the enteric nervous system (ENS) in humans and rodents and causes intestinal neuromuscular dysfunction in rats. Although infiltration of inflammatory cells in the myenteric plexus and neurodegeneration of enteric nerves are common features of patients suffering from functional intestinal disorders, the proof of a pathogenic link with HSV-1 is still unsettled mainly because the underlying mechanisms are largely unknown. In this study we demonstrated that following intragastrical administration HSV-1 infects neurons within the myenteric plexus resulting in functional and structural alterations of the ENS. By infecting mice with HSV-1 replication-defective strain we revealed that gastrointestinal neuromuscular anomalies were however independent of viral replication. Indeed, enteric neurons exposed to UV-inactivated HSV-1 produced monocyte chemoattractant protein-1 (MCP-1/CCL2) to recruit activated macrophages in the longitudinal muscle myenteric plexus. Infiltrating macrophages produced reactive oxygen and nitrogen species and directly harmed enteric neurons resulting in gastrointestinal dysmotility. In HSV-1 infected mice intestinal neuromuscular dysfunctions were ameliorated by *in vivo* administration of (i) liposomes containing dichloromethylene bisphosphonic acid (clodronate) to deplete tissue macrophages, (ii) CCR2 chemokine receptor antagonist RS504393 to block the CCL2/CCR2 pathway, (iii) Nω-Nitro-L-arginine methyl ester hydrochloride (L-NAME) and AR-C 102222 to quench production of nitrogen reactive species produced via iNOS. Overall these data demonstrate that HSV-1 infection makes enteric neurons recruit macrophages via production of a specific chemoattractant factor. The resulting inflammatory reaction is mandatory for intestinal dysmotility. These findings provide insights into the neuro-immune communication that occurs in the ENS following HSV-1 infection and allow recognition of an original pathophysiologic mechanism underlying gastrointestinal diseases as well as identification of novel therapeutic targets.

## Introduction

*Herpes simplex* virus type 1 (HSV-1) is counted among the most common viruses establishing chronic infections in humans (Straus, 2000). During primary infection, HSV-1 replicates in mucoepithelial cells of the mouth and throat. As neurotropic virus, it then gains access to sensory neurons and starts a latent infection, generally in the trigeminal ganglia (Swanson and McGavern, 2015). Throughout latency, HSV-1 expresses a unique pattern of RNAs, so-called LATs, persisting for the life of the host (Wilson and Mohr, 2012). The idea that HSV-1 is completely inactive during latency has been recently challenged since viral antigens expressed on latently infected human trigeminal ganglia mount a chronic inflammatory response impacting neuronal phenotype and function (van Velzen et al., 2013; Menendez et al., 2016). Latent HSV-1 periodically reactivates and travels through neuronal axons to the mucosal epithelium resulting in symptomatic or asymptomatic infections. Both infections similarly shed viral particles in the saliva and ensure transmission to immune competent individuals or secondary places in the same host (Straus, 2000; Kaufman et al., 2005; Miller and Danaher, 2008). By swallowing, infectious particles possibly reach the mucosa of the gastrointestinal tract and thereafter the enteric neurons embedded in the gut wall (Brun et al., 2010; Koyuncu et al., 2013). Indeed, HSV-1 DNA has been demonstrated in human nodose and celiac ganglia, neuronal structures innervating the gastrointestinal tract (Rand et al., 1984; Gesser and Koo, 1997). While in the central nervous system the HSV-1-driven inflammatory response has been linked to long-term neurologic deficits (Menendez et al., 2016) it is not clear how and at which extent HSV-1 infection impacts the functional integrity of the enteric neurons.

The innate and adaptive immune responses strongly cross-interact to control HSV-1 infection and to maintain the viral genome in the latent state. Pattern recognition receptors of the innate immunity recognize viral fragments and trigger secretion of interferons and cytokines which in turn shape specific adaptive immune responses to guarantee the immune surveillance in the infected tissue (Virgin et al., 2009; Koyuncu et al., 2013; Khoury-Hanold et al., 2016). The effectiveness of the immune response during HSV-1 infection strongly relies on the recruitment and differentiation of monocytes into tissue macrophages endowed with remarkable antiviral activities (Ellermann-Eriksen, 2005). Indeed, macrophages clear viral particles and apoptotic cells, release pro-inflammatory cytokines and chemokines to attract additional innate immune cells, limit viral replication, process, and present viral antigens to lymphocytes (Kodukula et al., 1999). Quite the opposite, infiltrating macrophages producing pro-inflammatory cytokines have been described also during latent HSV-1 infection and correlate with prolonged neuroinflammation in hypothalamus and hippocampus (White et al., 2016). Indeed, during chronic HSV-1 infection recruited immune cells impact the integrity of peripheral nerves either directly by damaging neurons harboring the virus or through the secretion of soluble factors that modify gene expression and survival of infected and non-infected cells (Kramer et al., 2003; Dosa et al., 2011).

We have recently described the first animal model of persistent HSV-1 infection in the enteric nervous system (ENS) thus providing the proof of concept that neurotropic viruses can reach enteric nerves and cause intestinal neuromuscular abnormalities (Brun et al., 2010). Since anti-inflammatory drugs have proved effective for treating patients suffering from gastrointestinal neuropathies, in this study we investigated the involvement of immune mediated mechanisms during the early phases of HSV-1-induced damage of the ENS. We demonstrated that HSV-1 triggers expression of monocyte chemoattractant protein-1 (MCP-1/CCL2) in enteric neurons thus recruiting macrophages in the myenteric ganglia. Infiltrating macrophages in turn produce reactive nitrogen species which cause structural and functional anomalies of the ENS resulting in gastrointestinal dysmotility.

## Materials and methods

### Viral stocks preparation and titration

Replicating HSV-1 strain SC16 and replication-defective HSV-1 mutants lacking the immediate-early genes coding for infected cell polypeptide (ICP)27 (HSV-1 IGR20 ΔICP27gJHE) or for ICP4, ICP27, and ICP22 (HSV-1 T0Z ΔICP4.ICP27.ICP22.UL41) were propagated on Vero cells (ATCC®CCL81™, American Type Culture Collection, VA, USA) or on 7b complementing cell lines, as previously described (Brun et al., 2010; Marconi et al., 2015). HSV-1 stocks were prepared in complete Dulbecco's modified Eagle's media (Gibco, UK) to obtain 1 × 10^8^ plaque-forming units (PFU)/mL.

### Mice infection and treatments

Wild type (WT) C57BL/6J male mice, 8–10 weeks old, were purchased from Envigo Laboratories (Udine, Italy). Animals were housed in a temperature-controlled environment (22 ± 2°C) under a 12 h light/dark cycle with food and water provided *ad libitum*. After 1 week acclimation period, mice were infected by intranasal (IN) instillation of HSV-1 strain SC16, 10^2^ PFU (10 μL final volume). Four weeks later, animals were inoculated via intragastric (IG) gavage (24 gauge, 9-cm catheter) with 1 × 10^7^ PFU of HSV-1 strain 16, HSV-1 IGR20 ΔICP27gJHE, HSV-1 T0Z ΔICP4.ICP27.ICP22.UL41, or equal volumes of Vero cell lysate (sham infection). The two-step protocol of HSV-1 administration was set to avoid high animal mortality rate (Brun et al., 2010). Following IG inoculum, mice were observed daily for signs of disease and sacrificed after 1, 2, or 3 weeks. Sham infected mice were sacrificed at matching time points; since the results were comparable, data were pooled together and reported as one sham infected animal group. As described, mice were injected intraperitoneally with rat anti-mouse CD4 purified monoclonal antibody (200 μg; clone GK1.5) for CD4 depletion; Nω-Nitro-L-arginine methyl ester hydrochloride (L-NAME, 25 mg/kg/day, Sigma, Italy) (De Visser et al., 2010), 5-[(4′-Amino-5′,8′-difluorospiro[piperidine-4,2′(1′H)-quinaxolin]-1-yl)carbonyl]-2-pyridinecarbonitrile hydrochloride (AR-C102222, a selective iNOS inhibitor, 30 mg/kg/day, Tocris Bioscience, UK) (LaBuda et al., 2006), CCR2 chemokine receptor antagonist (RS504393, 2 mg/kg/twice a day, Tocris Bioscience, UK) (Kitagawa et al., 2004), empty liposomes, or liposomes containing dichloromethylene bisphosphonic acid (clodronate), referred to as Clodrolip (Zeisberger et al., 2006). Anti-CD4 monoclonal antibody was produced by hybridoma (ATCC^®^ TIB207™) and purified using Protein G PLUS-Agarose (Santa Cruz Biotechnology, Italy). The initial dose of monoclonal antibody, L-NAME, AR-C102222 was administered immediately after IG HSV-1 inoculation and then for additional 7 days. The first dose of Clodrolip (1 mg/10 g body weight) was administered 5 days before IG HSV-1 inoculum and subsequently every 72 h (0.6 mg/10 g body weight). Sham infected mice received equal volumes of vehicle or empty liposomes, as appropriate. All experimental protocols were approved by the Animal Care and Use Committee of the University of Padova under license from the Italian Ministry of Health and followed the National and European guidelines for handling and use of experimental animals.

### HSV-1 infectivity and reactivation assays

HSV-1 infectivity was evaluated in the content of ileum. Briefly, mice were inoculated IG with HSV-1 strain SC16 or vehicle (sham infection) as described above and sacrificed 15 or 45 min later. The gut was carefully dissected, and content of the ileum was collected by flushing 5 ml sterile PBS. Samples were vigorously vortexed, centrifuged (3,000 rpm, 10 min) at 4°C, and equal volumes of the clarified supernatants were serially diluted and incubated with Vero cell monolayers. Vero cell cultures were monitored for up to 72 h to observe cytopathic effects. Cells were fixed and stained with crystal violet, and number of plaques was determined. Data are reported as percentage of the number of plaques obtained in Vero cells incubated directly with HSV-1 strain 16 (1 × 10^7^ PFU). HSV-1 presence was confirmed by PCR analysis on DNA extracted from Vero cells.

### Intestinal whole mount preparation and staining

At the time of sacrifice, 8 cm segment of the distal ileum was flashed with PBS, filled with 4% PFA and submerged in the same fixative for 1 h at 22°C. Tissues were then washed in PBS (3 × 10 min) and stored at 4°C (Brun et al., 2013). Whole mounts were prepared from 1 cm long specimen under a dissecting microscope (Zeiss, Germany) by peeling off the longitudinal muscle layer containing the myenteric plexus (LMMP). LMMP were gently stretched, pinned down on a wax support, washed twice with PBS containing 0.5% Triton-X100, incubated in blocking buffer (2% bovine serum albumin, 0.5% Triton-X100 in PBS) and then stained at 4°C for 16 h with primary antibody (Table 1). Fluorescent labeled secondary antibodies (Table 1) were used to detect immune-complexes using Leica TCSNT/SP2 confocal microscope.

**Table 1:** Primary and secondary antibodies used in the study.

**Antigen (host)**	**Clone**	**Source**	**Application**
**Primary antibodies**			
βIII-Tubulin (rabbit)	Polyclonal	Couvance	WM
Peripherin (rabbit)	Polyclonal	Millipore	WM
S100β (rabbit)	EP1576Y	Millipore	WM
ChAT (rabbit)	Polyclonal	Gene Tex	WB
nNOS (rabbit)	Polyclonal	Invitrogen	WB
CCL2 (rat)	ECE.2	Abcam	IHC
β-Actin (mouse)	AC-15	Sigma-Aldrich	WB
CD3 (rat)	17A2	eBioscience	IHC, FC
CD4 (rabbit)	50134-R001	Sino Biological Inc.	FC
CD8 (rabbit)	orb1269	Biorbyt Ltd	FC
IFNγ (rat)	XMG1.2	eBioscience	FC
IL4 (rat)	11B11	eBioscience	FC
F4/80 (rat)	CI:A3-1	Abcam	FC
CD11b (rabbit)	EPR1344	Abcam	IHC, FC
CD19 (rabbit)	C1C3	Gene Tex	FC
NK1.1 (rabbit)	PK136	Gene Tex	FC
gD (mouse)	1-I-9	Abcam	WB
gC (mouse)	3G9	Abcam	WB
**Antigen (host)**		**Source**	**Application**
**Secondary antibodies**			
Anti-rabbit (goat) PE		Chemicon	WM, FC, IHC
Anti-rat (rabbit) FITC		Invitrogen	IHC, FC
Anti-rabbit (goat) HRP		Sigma-Aldrich	WB, IHC
Anti-mouse (goat) HRP		Sigma-Aldrich	WB
Anti-rat (rabbit) HRP		Sigma-Aldrich	IHC
Anti-rabbit (goat) APC		Chemicon	FC
Anti-rat (rabbit) PE		Sigma-Aldrich	FC

*WB, western blot; WM, whole mount; IHC, immunohistochemistry; FC, flow cytometry*.

### Isolation of longitudinal muscle myenteric plexus

At the sacrifice, the small intestine was aseptically removed, washed in oxygenated Krebs solution (126 mM NaCl, 2.5 mM KCl, 25 mM NaHCO3, 1.2 mM NaH2PO4, 1.2 mM MgCl2, 2.5 mM CaCl2, pH 7.2) and cut in pieces of 1 cm length. Intestinal segments were placed on a sterile glass rod and a small incision was created in the longitudinal muscle by gently rub the edge of the forceps. The LMMP were then peeled off under a dissecting microscope (Zeiss, Germany) and immediately snap-frozen in liquid nitrogen or subjected to enzymatic digestion to isolate macrophages, lymphocytes, or enteric neurons.

### Isolation and culture of enteric neurons

Freshly obtained LMMP were rinsed three times in Krebs, minced with scissors and digested in 1.3 mg/ml collagenase type II (Sigma) and 0.3 mg/ml bovine serum albumin at 37°C for 15 min. Cells were then co-cultured with Vero cells or seeded on laminin and poly-D-lysine (all from Sigma) coated coverslips in Neurobasal A media containing B-27 supplement, 1% fetal bovine serum, 10 ng/mL nerve growth factor (NGF, BioLegend, Italy), and penicillin/streptomycin. Half of the cell media was replaced every 2 days with fresh complete growth media (Brun et al., 2015). As indicate, at the fifth day in culture, neurons were incubated with HSV-1 for 36 h and then fixed in PFA 4% for 20 min at room temperature and subjected to immunocytochemistry following the same protocol described in 2.4. Alternatively, enteric neurons were challenged with HSV-1 inactivated by exposure to 20 Joules of ultraviolet (UV) light for 10 min at maximal power output.

### Nucleic acid extraction and analysis

Vero cells were homogenized in digestion buffer and DNA was extracted using “Tissue DNA Extraction Kit” (Millipore, Milan, Italy). LMMP were homogenized using a Retsch MM300 mixer and RNA was extracted using SV total RNA isolation system (Promega, Italy) following manufacturer's protocol. Contaminating DNA was removed by DNase I treatment (Promega). Complementary DNA (cDNA) was generated as previously described (Brun et al., 2013). To study HSV-1 reactivation and replication cycle, 5 μL of cDNA were subjected to semi-quantitative PCR. Amplification products were separated on agarose gel and visualized by Nancy-520 DNA gel stain (Sigma, Milan, Italy) using UV illuminator. Quantitative PCR was performed using the ABI Prism 7700 Sequence Detection System (PerkinElmer, Monza, Italy) and iTaq Universal SYBR Green One-Step Kit (Bio-Rad Laboratories, CA, USA). Specific oligonucleotides were designed in two adjacent exons (Universal Probe Library Assay Design Center, Roche Applied Science) and are listed in Table 2. Data were normalized to 18S ribosomal RNA (Rn18S) and plotted as mean fold changes.

**Table 2:** Oligonucleotides and PCR conditions.

**Oligonucleotide**	**Sequence**	**Tm (°C)**
tk	Fw 5′-tagcccggccgtgtgaca-3′ Rv 5′-cataccggaacgcaccacacaa	60
LATs	Fw 5′-gacagcaaaacaataaggg-3′ Rv 5′-acgagggaaaacaataaggg-3′	60
ICP4	Fw 5′-atgacggggacgagtacgac-3′ Rv 5′-acgacgaggacgaagaggat-3'	56
VP16	Fw 5′-tgcgggagctaaaccacatt-3′ Rv 5′-tccaacttcgcccgaatcaa-3′	60
gB	Fw 5′-ggctccttccgattctcc-3′ Rv 5′-ggtactcggtcaggttggtg-3′	60
gC	Fw 5′-ccaaacccaagaacaacacc-3′ Rv 5′-tgttcgtcaggacctcctct-3′	60
β-actin	Fw 5′-aggaagccactctagggagc-3′ Rv 5′-agaacagagtgagcgggaga-3′	60
Ccl2	Fw 5′-gcctgctgttcacagttgc-3′ Rv 5′-caggtgagtggggcgtta-3′	60
Ccr2	Fw 5′-acctgtaaatgccatgcaagt-3′ Rv 5'-tgtcttccatttcctttgatttg-3′	60
Rn18S	Fw 5′-tcaagaacgaaagtcggagg-3′ Rv 5′-ggacatctaagggcatca-3′	60

*Fw, forward; Rv, reverse; Tm, melting temperature*.

### Histological evaluation

Mice were sacrificed at the specified time. Specimens of ileum (5-10 cm long segments starting from the ileocecal valve) were fixed in 10% neutral buffered formalin for 24 h, embedded in paraffin and sectioned at 5 μm thickness. Six representative sections per mouse were subjected to Hematoxylin and Eosin (H&E) staining. A minimum of 10 independent fields per animal were examined using Leica microscope equipped with a digital camera.

### Immunohistochemistry

Four μm thick sections were obtained from paraffin embedded samples of ileum, deparaffinized and rehydrated (xylene 5 min; ethanol 100, 95, 70%, 1 min each), using standard procedures (Brun et al., 2013). Slices were exposed to 10% H_2_O_2_ to block endogenous peroxidase activity, treated with citrate buffer (pH 9) for antigen retrieval, and finally incubated with universal blocking solution (Lab Vision Corporation, CA, USA). Tissue sections were then exposed to proper antibody (Table 1) for 1 h at 22°C. The Dako Envision+ System-HRP labeled Polymer Detection system (Dako, CA, USA) was used, with either 3,3′ diaminobenzidinetetrahydrochloride (DAB) as chromogenic substrate. Sections were H&E counterstained and observed using conventional microscopy. As negative control, sections were stained either by isotype-matched antibody of inappropriate specificity or by omitting the primary antibody.

### Neurological assessment

Neurological integrity of each mouse was assessed twice a week by a blinded observer using a validated scoring system (Garcia et al., 1995). Six tests were performed to evaluate sensorimotor functions: (1) spontaneous activity; (2) symmetry in the movement of the four limbs; (3) forepaw outstretching; (4) climbing; (5) body proprioception; and (6) response to vibrissae touch.

### *In vivo* gastrointestinal transit

Mice were administered IG with fluorescein-isothiocyanate dextran (70,000 MW; 6.25 mg/mL in PBS; 100 μL/mice; MP Biomedicals LLC, CA, USA). Animals were sacrificed 60 min later and the entire bowel was gently removed. Small intestine was divided in 8 equal segments whereas stomach, cecum and colon were examined separately. Luminal contents were collected and clarified by centrifugation (10,000 × g, 15 min, 4°C). Fluorescence analysis was performed at 494/521 nm (Hitachi F2000; Hitachi, Tokyo, Japan). Gastric emptying was calculated as the percentage of FITC-dextran left in the stomach compared with the total amount of fluorescence in the gastrointestinal tract. Intestinal transit was reported as the geometric center of distribution of the fluorescent probe throughout the ileum (Brun et al., 2013).

### Distal colonic transit measurements

Distal colonic transit was assessed in fasted mice briefly anesthetized with isoflurane (<1 min; Merial, France). As previously described (Martínez et al., 2002), a single 2-mm glass bead was inserted into the distal colon at 2 cm from the anus. After bead insertion, mice were placed individually in plastic cages lined with white paper to aid visualization of bead expulsion. Mice readily regained consciousness showing normal behavior. Distal colonic transit was determined to the nearest 0.1 min by monitoring the time required for the expulsion of the glass bead (bead retention).

### *Ex vivo* contractility studies

*Ex vivo* contractility studies were performed as previously described (Brun et al., 2013) on full-thickness distal ileum segments. Contractions were evoked by electrical field stimulation (EFS; 2–50 Hz, 1-ms pulse duration, 10-s pulse trains, 60 V) using platinum electrodes connected to a S88 stimulator (Grass Instrument, MA, USA). When indicated, EFS-induced non-adrenergic, non-cholinergic (NANC) relaxation responses were obtained using increasing frequencies of stimulation (2–40 Hz) in presence or absence of L-NAME 100 μM.

### Immunoblot analysis

LMMP were lysed in RIPA buffer (150 mM NaCl, 50 mM Tris-HCl, 0.25% sodium deoxycholate, 0.1% Nonidet P-40, 100 μM NaVO4, 1 mM NaF) containing a mixture of protease inhibitors (0.5 mM EDTA, 0.1 mM PMSF, 1 μM leupeptin, 150 nM aprotinin) (Brun et al., 2013). Samples were incubated 30 min at 4°C. Particulate material was removed by centrifugation (15,000 × g, 5 min at 4°C) and protein concentration was determined in the supernatants using the bicinchoninic acid assay (Thermo Scientific, MA, USA). Proteins (40 μg/line) were fractionated through SDS-PAGE gel and immobilized onto PVDF membrane (Bio-Rad Laboratories). Unspecific bindings were blocked for 1 h in 5% non-fat dry milk dissolved in PBS and added with 0.05% Tween20. PVDF membranes were then probed with specific antibodies (Table 1). Immune-complexes were revealed using horseradish peroxidase (HRP)-conjugated secondary antibodies (Sigma-Aldrich, Italy; Table 1) and enhanced chemiluminescent substrate (ECL, Millipore, Italy). Images were captured using Hyper Film MP (GE Healthcare, Italy). Antibody against mouse β-actin (Sigma) was used as loading control. Densitometric analysis was performed using the ImageJ software (US National Institutes of Health).

### Serological assay

Blood samples (~1.0 ml) collected from sham, IN and IG HSV-1 infected mice were incubated at room temperature for 60 min to allow clotting. Serum was then separated by centrifugation (2,000 rpm, 10 min) and stored at −20°C until it was used to determine the presence of anti-HSV-1 IgG using commercially available ELISA (Mouse/Rat HSV-1 IgG ELISA; Calbiotech, CA, USA) following manufacturer's protocol.

### Isolation of mononuclear cells

To isolate lymphocytes, spleens were dissociated by forcing tissue through a 100 μm metallic mesh. To obtain lymphocytes and macrophages from LMMP, freshly isolated ileum was cut in small pieces and LMMP peeled off. Then, LMMP were finely minced using scissors and dissociated by incubation for 10 min at 37°C with collagenase type II from Clostridium histolyticum (10 mg/ml), dispase (60 μg/ml) and DNase I (10 μg/mL, all purchased from Sigma). Tissue debris was filtered through a cell strainer. Cells were collected (900 × g for 5 min), purified by density gradient centrifugation through Ficoll-Hypaque (Sigma) and immediately stained for flow cytometry analysis or cultured for 16 h at 37°C with or without UV-inactivated HSV-1.

### Flow cytometry analysis

Macrophages and lymphocytes (10^6^/mL) freshly dissociated as above described were incubated for 20 min in PBS containing 2% w/v bovine serum albumin and stained for 30 min at 4°C with proper antibodies (Table 1). For intracellular cytokine staining, cells were subsequently incubated for 30 min at room temperature in fixation/permeabilization buffer (eBioscience, Italy) containing the proper antibody. Cells were then washed, and fluorescent signal was recorded using BD FACSCanto™ Flow Cytometry (BD Bioscience, Italy). The results were analyzed using the WinMDI 2.9 (Windows Multiple Document Interface for Flow Cytometry) program.

### Detection of intracellular free radicals

Macrophages (10^6^/mL) freshly dissociated from LMMP as above described were loaded for 30 min at 37°C with 10 μM 2′,7′-dichlorodihydrofluorescein diacetate (H_2_DCFDA; Molecular Probes, Italy) to detect intracellular ROS or with 5 μM 4,5-diaminofluorescein (DAF-FM) diacetate (Molecular Probes) to reveal intracellular NO. At the end of incubation, cells were washed twice, stained with the proper antibodies, and analyzed by flow cytometry.

### CCL2 detection by ELISA

LMMP were homogenized in PBS (1:10 wt/vol) supplemented with protease inhibitors (1 mmol/L phenylmethylsulfonyl fluoride, 10 μg/mL aprotinin, 10 μg/mL leupeptin) and centrifuged (10,000 × g, 10 min at 4°C). Clarified supernatant was assessed for CCL2 protein levels using commercially available kit (eBioscience), following manufacturer's protocol. Optical densities were registered at 450 nm using a microplate reader (Sunrise, Tecan; Switzerland). The sensitivity of the assay was 15 pg/ml.

### Statistical analysis

Results were given as mean ± SEM, except for distribution of the fluorescent probe in *in vivo* gastrointestinal transit, which was presented as median ± SEM. Differences in the mean for the different experimental groups was tested using one-way ANOVA analysis followed by Bonferroni multicomparison *post-hoc* tests. The levels of statistical significance are shown in figure legends. A *P*-value of 0.05 or less was considered statistical significant. Statistical analyses were performed using GraphPad Prism 3.03 software (GraphPad, San Diego, CA).

## Results

### HSV-1 infects neurons of the enteric nervous system

In a previous study (Brun et al., 2010) we reported that HSV-1 establishes a latent infection in the myenteric ganglia of rats. Indeed, following intragastric inoculation of HSV-1, we found latency-associated and early viral gene transcripts in neurons of the ileum but no expression of late viral genes, suggesting an abortive replication of HSV-1. Despite the ease at which neurons can be studied in rats, the animal model has many limitations including species-specific reagents. Therefore, we moved to develop a murine model of HSV-1 infection of the ENS.

**Figure 1:**
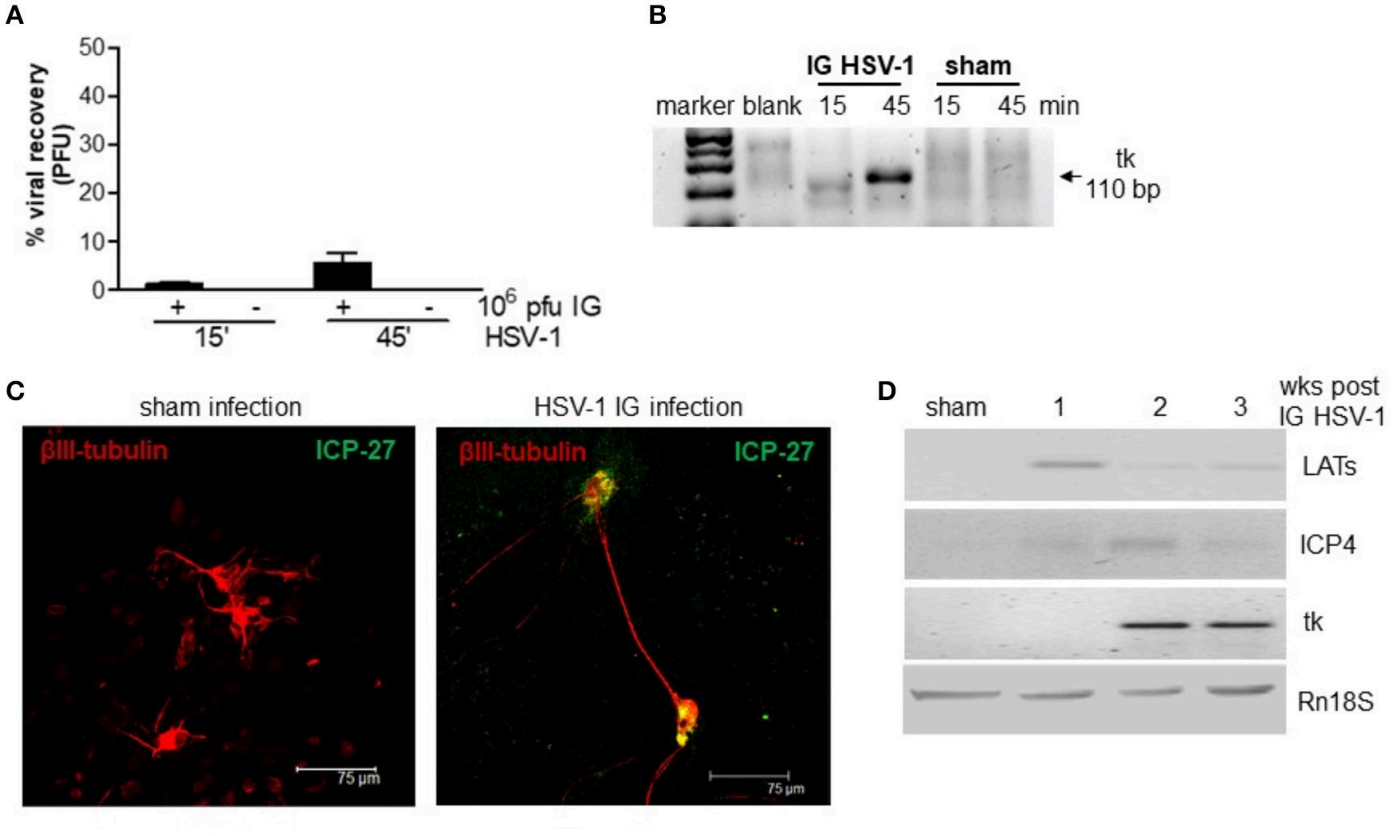
HSV-1 infection of the ENS. **(A)** The content of the ileum was collected from mice at 15 and 45 min post-IG inoculum with HSV-1 strain 16 (1 × 10^7^ PFU). Intestinal content was co-cultured on Vero cells and plaques were counted. The number of plaques derived from Vero cells incubated with HSV-1 strain SC16 (1 × 10^7^ PFU) but not injected in mice was taken as 100%. Data are represented as mean ± SEM of three separated experiments. *n* = 4 mice per group. **(B)** Total DNA was isolated from Vero cells exposed for up to 72 h to content of ileum obtained from mice inoculated IG with HSV-1 or sham infected. DNA was subjected to PCR using HSV-1 tk specific primers. Representative picture of one out of three agarose gel is reported. bp: base pairs. **(C)** Primary enteric neurons were infected with HSV-1, fixed 36 h later, and probed with anti-β-III-tubulin (red) and anti-ICP-27 (green) antibodies. Cells were imaged using a confocal microscope. Representative images are shown. **(D)** One, two, and three weeks post-IG inoculum of HSV-1 strain SC16, total RNA was purified from LMMP and semi-quantitative PCR was performed to evaluate the expression of HSV-1 LATs and early (ICP4, tk) mRNA transcripts. A representative experiment is shown. Rn18S was used as extraction and loading control. Sham: mice IG inoculated with Vero cell lysate.

We first ascertained the ability of HSV-1 to retain infectivity following the IG inoculum. Mice were inoculated IG with HSV-1 strain SC16 or equal volume of Vero cell lysate (sham infection) and the ileum content was recovered after 15 and 45 min, vigorously mixed, and centrifuged. The clarified supernatants were incubated on Vero cell monolayers. As reported in Figure 1, contents of ileum collected 15 and 45 min post IG viral inoculum revealed respectively 1 and 10% of injected PFU as demonstrated by the classical plaque titration assays (Figure 1A). The presence of HSV-1 in Vero cells was confirmed by semi-quantitative PCR analysis (Figure 1B). Samples obtained from mice subjected to sham infection did not produce cytopathic effects on Vero cells.

To demonstrate the ability of HSV-1 strain SC16 to infect murine enteric neurons, cultured primary murine enteric neurons were infected with HSV-1 strain SC16 (MOI 1:1). Cells were fixed 36 h later and then stained with anti-ICP-27 antibody. As shown in Figure 1C, ICP-27 immunoreactivity is evident in β-III-tubulin positive neurons, thus confirming the ability of HSV-1 to infect and replicate in mouse enteric neurons.

In the attempt to define the nature of HSV-1 infection in the murine myenteric plexus, we isolated total RNA from LMMP preparations of IG infected mice. HSV-1 latency-associated transcripts (LATs) and mRNA transcripts of the early gene ICP4 were detected to varying extents in the LMMP of animals at 1, 2, and 3 weeks after IG administration but were undetectable in mice inoculated with Vero cell lysate (sham infection; Figure 1D). HSV-1 thymidine kinase (tk) mRNA were evident in the LMMP only at 2 and 3 weeks post-IG inoculum (Figure 1D). Since we did not observe expression for late genes (VP16, gB, gC) or VP16, gD, and gC proteins (data not shown) we concluded that HSV-1 persistently infected the murine myenteric plexus and carried on an abortive viral replication, as previously reported in the rat model (Brun et al., 2010).

### Orogastric HSV-1 inoculum causes intestinal dysmotility in mice

**Figure 2:**
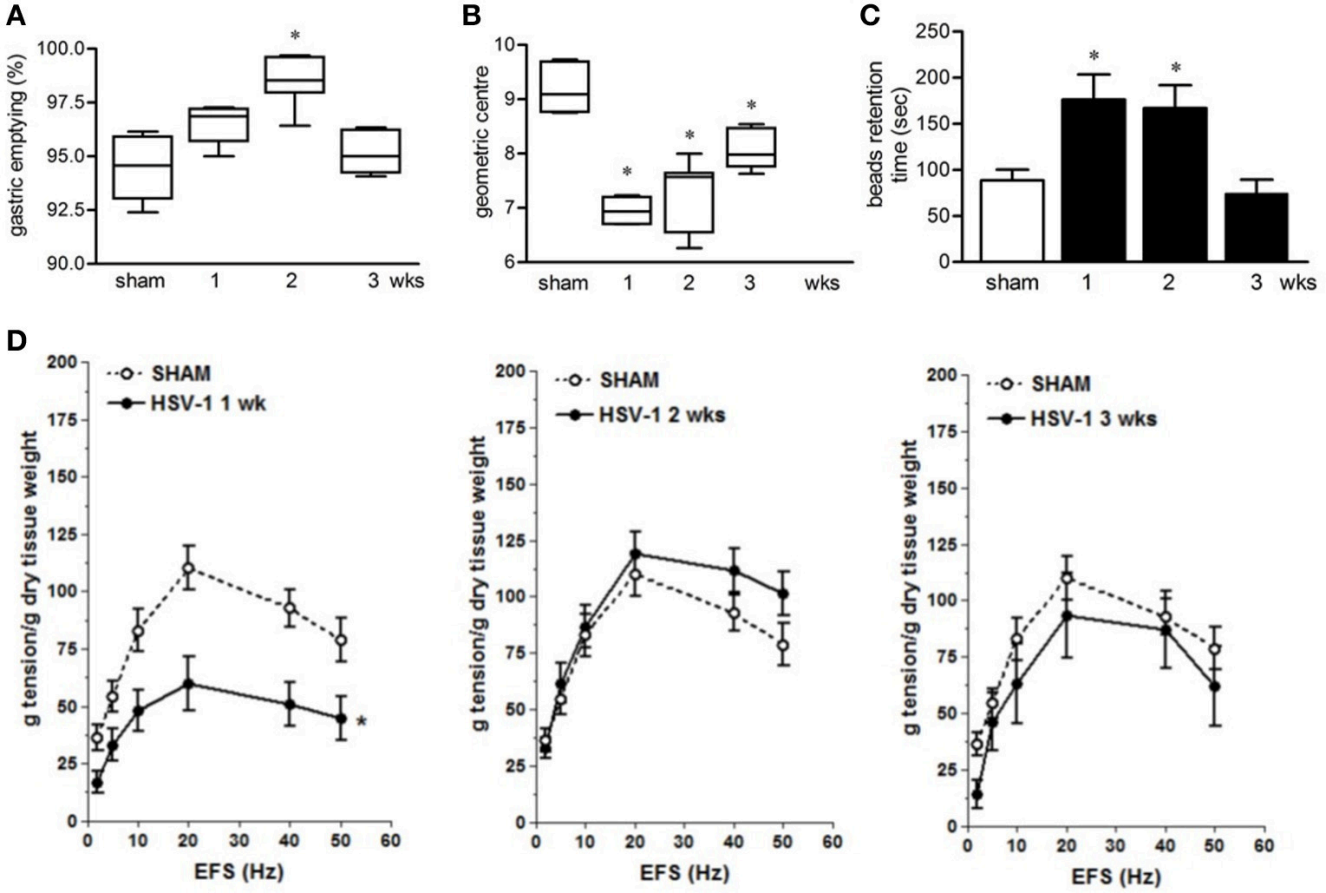
Gastrointestinal dysmotility in HSV-1 infected mice. **(A)** One, two, and three weeks post-IG infection with replicating HSV-1 strain SC16, mice were injected with non-absorbable FITC-labeled dextran. Sixty minutes later mice were sacrificed. Gastric emptying was calculated as the percentage of probe retained into the stomach compared with the total amount of fluorescence in the gastrointestinal tract. **(B)** Distribution of FITC-labeled dextran was determined in the intestine. Intestinal transit was reported as the geometric center of distribution of the fluorescent probe throughout the ileum. **(C)** Time (seconds, sec) required for expulsion of a glass bead inserted into the rectum. **(D)** Electrical field stimulation (EFS)-elicited contractions in segments of ileum from sham and IG HSV-1 infected mice. Data are represented as mean ± SEM. *n* = 6–12 mice per group. ^*^Denotes *P* < 0.01 compared to sham infected mice.

Infection with HSV-1 strain 16 or replication-defective mutants did not produce signs of central or peripheral neurological deficit in mice (data not shown) but resulted in time-dependent motility anomalies in different intestinal segments. IG inoculum of HSV-1 strain SC16 caused faster gastric emptying, delayed intestinal transit and slower colonic motility as compared to sham infected animals (Figures 2A–C). The intestinal neuromuscular contractility was transiently impaired. Thus, electrical field stimulation (EFS)-elicited contractions in isolated segments of ileum was significantly reduced at the first week of infection compared to those from sham infected mice and was almost completely restored at the second and third week post-infection (Figure 2D). No alterations in gastrointestinal transit and EFS-induced contractility were detected in sham infected mice.

### HSV-1 infection causes neuroplastic changes of the ENS

**Figure 3:**
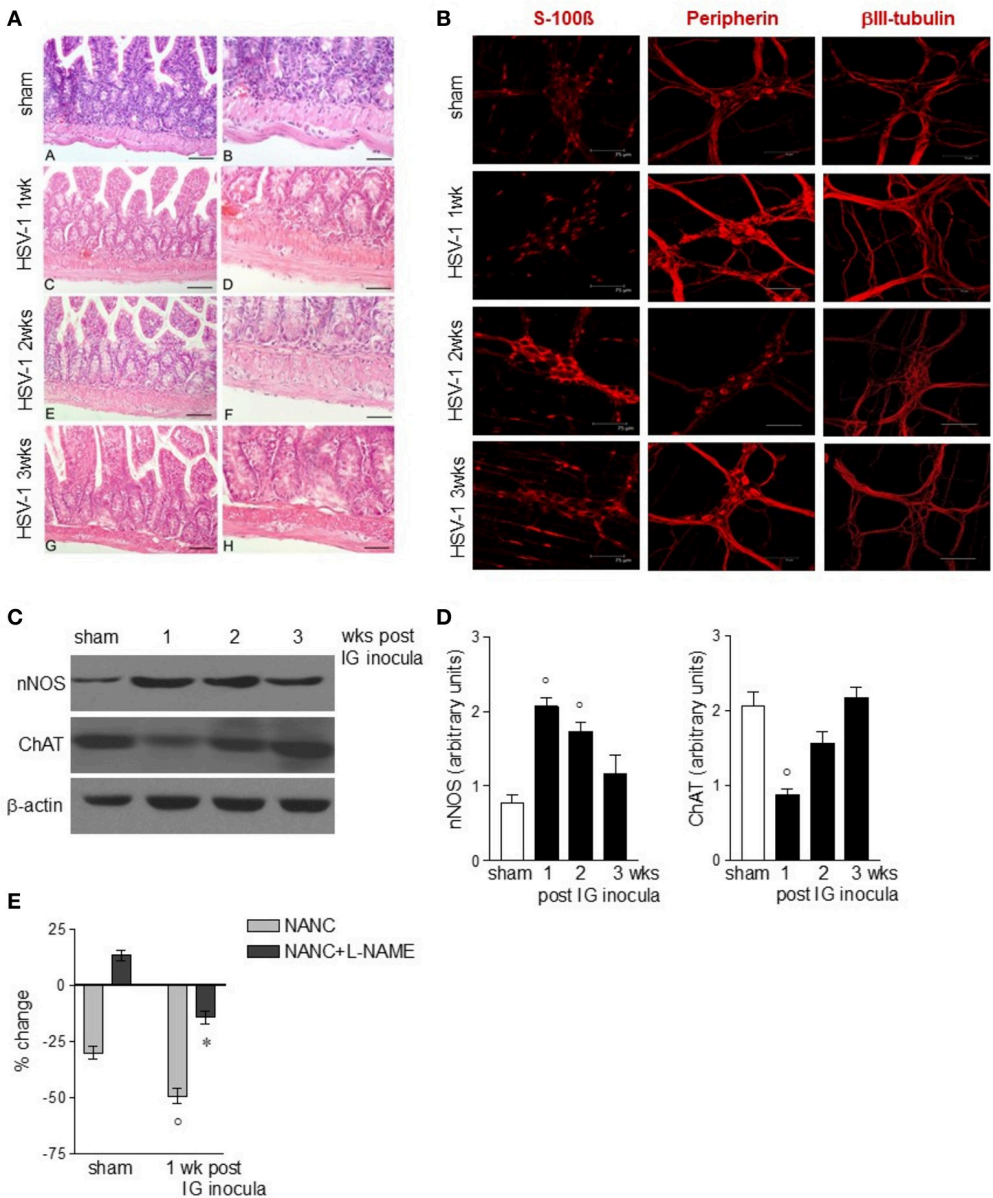
Alterations in myenteric plexus during HSV-1 infection. **(A)** One, two, and three weeks post-IG infection with HSV-1 strain SC16, distal ileum was removed and fixed in neutral buffered formalin, embedded in paraffin, and subjected to H&E staining. Representative images are presented. Scale bars: 40 μm in (left panels); 20 μm in (right panels). **(B)** Samples of distal ileum were fixed to obtain whole mount preparations. Immunofluorescence analysis for S-100β (glial marker), neurofilament peripherin, and neurotubules βIII-tubulin was performed. Scale bars: 75 μm. Representative images of three separate experiments. **(C)** Western Blot analysis of nNOS and ChAT in protein extracts from LMMP of sham and IG HSV-1 infected mice. β-actin was used as loading control. Images representative of three different blots with similar results are reported. **(D)** Protein signals were quantified using ImageJ and reported as arbitrary units. **(E)** Non-adrenergic, non-cholinergic (NANC) responses of segments of ileum obtained from sham and 1-week HSV-1 infected mice evoked by EFS in absence or presence of L-NAME. Percent change is expressed as change in grams of active tension before EFS-induced relaxation. Data are represented as mean ± SEM of at least two separate experiments. °Denotes *P* < 0.05 compared to sham infected mice. ^*^Denotes *P* < 0.02 compared to mice at 1 week of infection in absence of L-NAME.

The functional studies revealed compromised gastrointestinal motor function. Since the ENS controls intestinal motility we next evaluated the integrity of the neuroglial network in the gut wall of mice subjected to IG HSV-1 inoculum. Although the histological examination of HSV-1 infected mice revealed conserved architecture in the small intestine (Figure 3A), immunofluorescence studies on whole mount preparations of LMMP demonstrated abnormalities in neurons and glial cells (Figures 3B–D). Immunoreactivity of the glial marker S-100β, a Ca^2+^-modulated protein implicated in intracellular and extracellular regulatory activities, was increased mainly at the second and third week post-IG HSV-1 inoculum (Figure 3B), indicating activation of enteric glial cells. Though immunoreactivity of peripherin and βIII-tubulin enhanced 1-week post-IG HSV inoculum, the structural integrity of neurofilaments was overall preserved (Figure 3B). However, the neurochemical code in the ENS significantly changed following HSV-1 infection. Neuronal nitric oxide synthase (nNOS) protein expression increased in the LMMP of HSV-1 infected mice. Expression of choline acetyltransferase (ChAT) decreased 1-week post-HSV-1 inoculum but regained levels comparable with sham infected mice at later time points (Figures 3C,D). Considering the impact of nitric oxide as inhibitory neurotransmitter on intestine, we then verified the functional consequences of increased nNOS expression in the myenteric plexus of HSV-1 infected mice. In agreement with previous reports (Serio et al., 2003), at 1-week post-infection non-adrenergic, non-cholinergic evoked relaxation in explants of ileum was abolished by the NOS inhibitor N_ω_-Nitro-L-arginine methyl ester hydrochloride (L-NAME), indicating its nitrergic origin (Figure 3E). Thus, the increased expression of nNOS in the myenteric plexus of HSV-1 infected mice amplified the nitrergic effect in intestinal contractility, accounting for the delayed gastrointestinal transit (see Figure 2B). Altogether these data support the view that, following IG inoculum, HSV-1 induces neuroplastic changes in neuronal circuits of the gut leading to dysmotility.

### Gastrointestinal neuromuscular dysfunctions are independent of viral replication

**Figure 4:**
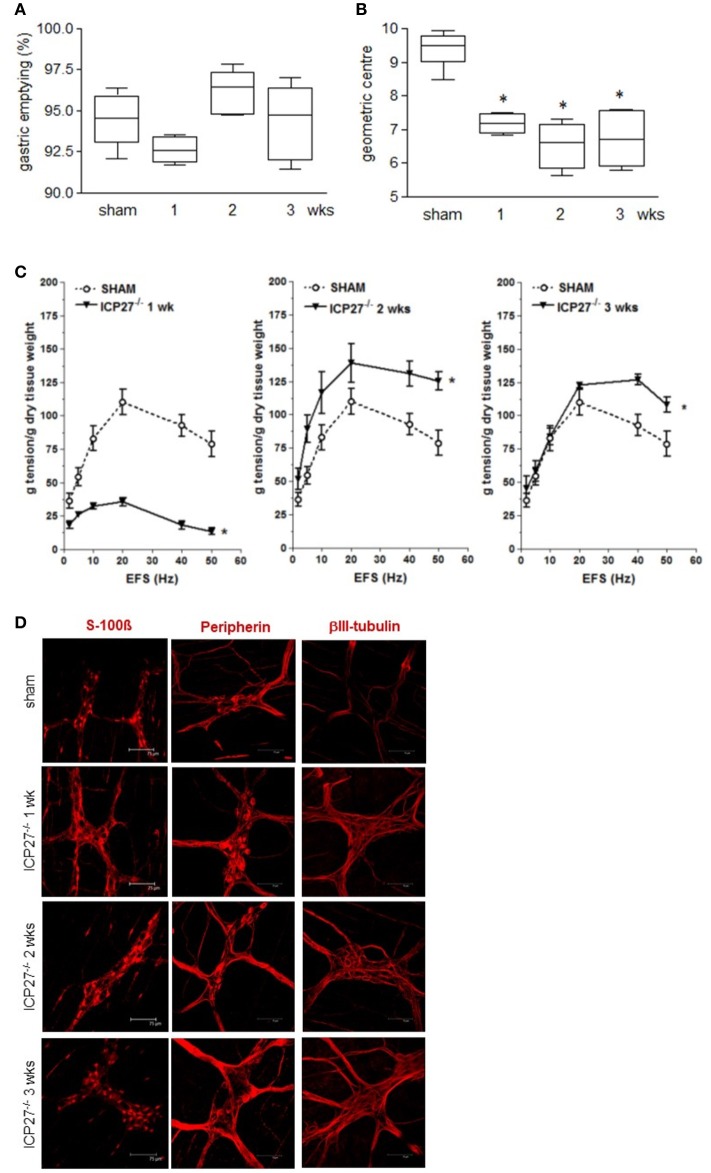
Persistence of gastrointestinal alterations in mice infected with HSV-1 replication-defective strain **(A)** Mice were injected IG with HSV-1 IGR20 ΔICP27gJHE (ICP27^−/−^) replication-defective strain. Mice received non-absorbable FITC-labeled dextran 1, 2, and 3 weeks post-IG inocula. Sixty minutes later mice were sacrificed, and gastric emptying was calculated as the percentage of probe retained into the stomach compared with the total amount of fluorescence in the gastrointestinal tract. **(B)** Distribution of FITC-labeled dextran was determined in the intestine. Intestinal transit was reported as the geometric center of distribution of the fluorescent probe throughout the ileum. **(C)** EFS-elicited contractions in segments of ileum of sham and IG HSV-1 ICP27^−/−^ infected mice. Data are represented as mean ± SEM. *n* = 6 mice per group. ^*^Denotes *P* < 0.01 compared to sham infected mice. **(D)** Immunofluorescence analysis for S-100β, peripherin, and βIII-tubulin on whole mount preparations of ileum of HSV-1 ICP27^−/−^ infected mice. Scale bars: 75 μm. Representative images of three separate experiments.

To dissect the factors responsible for ENS neuroplasticity and gastrointestinal dysmotility, we first verified whether HSV-1 replication is mandatory for the onset of neuronal alterations. Mice were infected with two engineered HSV-1 strains deleted of immediate early genes essential to initiate viral replication (Marconi et al., 2010). As shown in Figures 4A–C, the IG inoculum with replication-defective HSV-1 IGR20 ΔICP27gJHE (ICP27^−/−^) induced gastrointestinal contractile dysfunctions resembling those observed following infection with HSV-1 strain SC16 and reported in Figure 2. At the same time, the replication-defective virus caused structural abnormalities of the ENS (Figure 4D). Infection with the other replication-defective HSV-1, T0Z ΔICP4.ICP27.ICP22.UL41 (Krisky et al., 1998), caused similar functional and structural anomalies of the ENS (data not shown). These data indicate that the neuromuscular dysfunctions observed following IG HSV-1 inoculum are not merely due to viral replication, but most likely result from host-mediated immune mechanisms.

### Adaptive immune responses do not contribute to intestinal neuromuscular dysfunction

**Figure 5:**
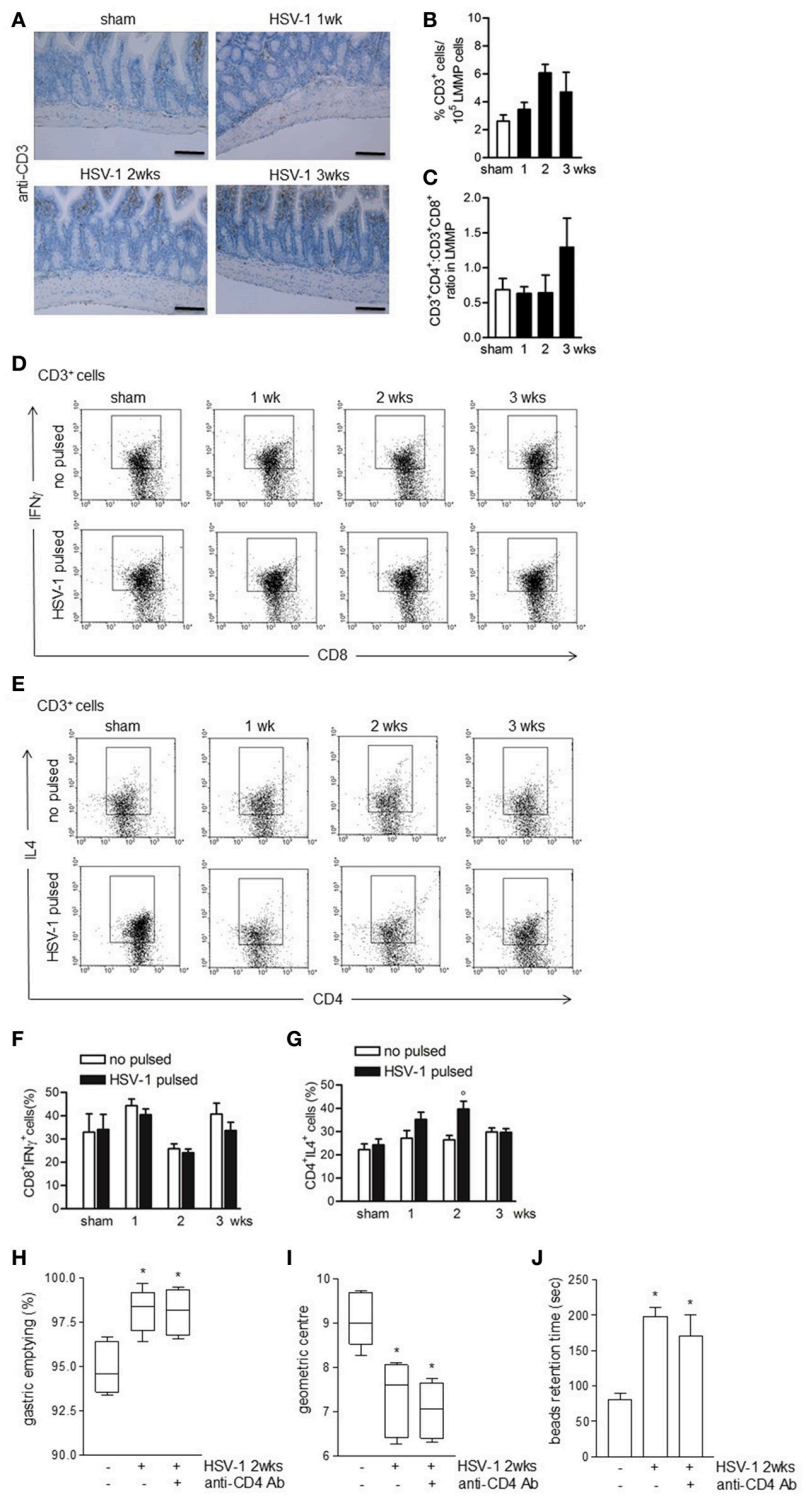
Weak adaptive immune response in the LMMP following HSV-1 administration. **(A)** Sections of ileum obtained from sham or HSV-1 strain SC16 infected mice were subjected to immunohistochemistry for CD3. Scale bars: 40 μm. Representative images of three separate experiments. **(B)** Freshly collected LMMP were digested and the resulting cell suspension were labeled with anti-CD3 antibody and analyzed by flow cytometry. CD3^+^ cells were expressed as percentage of 10^5^ collected events. **(C)** Cell suspensions obtained from LMMP as previously described were labeled with anti-CD3, anti-CD4 or anti-CD8 antibodies and analyzed by flow cytometry. CD4:CD8 ratio of 1 × 10^5^ CD3^+^ cells was calculated. **(D,E)** Cell suspensions obtained from LMMP were incubated for 16 h in presence or absence of UV-inactivated HSV-1 strain SC16. Cells were then collected, labeled with anti-CD3, anti-CD8 and anti-IFNγ or anti-CD3, anti-CD4 and anti-IL4 antibodies and analyzed by flow cytometry in 5 × 10^4^ collected events. Representative images of at least five separate experiments are reported. **(F,G)** Percentages of fluorescence as reported in **(D,E)** were graphed. *N* = 5 mice per group. °Denotes *P* < 0.05 compared to no HSV-1 pulsed cells at the same time point. **(H)** Mice infected with HSV-1 strain 16 were intraperitoneally injected with rat anti-mouse CD4 purified monoclonal antibody (anti-CD4 Ab). Two weeks post-IG infection, mice received IG non-absorbable FITC-labeled dextran. Sixty minutes later mice were sacrificed. Gastric emptying was calculated as the percentage of probe retained into the stomach compared with the total amount of fluorescence in the gastrointestinal tract. **(I)** Distribution of FITC-labeled dextran was determined in the intestine. Intestinal transit was reported as the geometric center of distribution of the fluorescent probe throughout the ileum. **(J)** Time (seconds, sec) required for expulsion of a glass bead inserted into the rectum. Data are represented as mean ± SEM. *n* = 6–9 mice per group. ^*^Denotes *P* < 0.05 compared to sham infected mice.

To investigate the role of immune cell activation on gastrointestinal neuromuscular dysfunctions we at first examined lymphocyte recruitment. Four weeks following IN HSV-1 inoculum, circulating anti-HSV-1 IgG and HSV-1 reactive spleen CD3^+^ lymphocytes were evident (data not shown). However, in the LMMP, the distribution and percentage of CD3^+^ cells, the CD4^+^:CD8^+^ ratio, and the percentage of CD3^+^CD8^+^IFNγ^+^ cells were unaffected by IG HSV-1 inoculum (Figures 5A–D,F). HSV-1 responsive CD3^+^CD4^+^IL4^+^ cells were revealed at the second week following IG HSV-1 inoculum (Figures 5E,G). Moreover, the percentage of CD3^−^CD19^+^ B lymphocytes and CD3^−^NK1.1^+^ NK T cells did not change following IG HSV-1 inoculum as compared with sham infected mice (data not shown).

To ascertain the role of HSV-1 activated CD3^+^CD4^+^ cells on gastrointestinal neuromuscular dysfunctions, infected mice received an intraperitoneal bolus of monoclonal anti-CD4 antibody 1 day following IG HSV-1 administration and were sacrificed at the second week post-IG infection. Depletion of CD4^+^ cells did not correct the gastrointestinal and colonic dysmotility (Figures 5H,J) suggesting that the HSV-1-driven CD3^+^CD4^+^ cells recruitment in the LMMP does not account for the observed gastrointestinal neuromuscular dysfunctions.

### Macrophages infiltrating the LMMP damage the ENS

**Figure 6:**
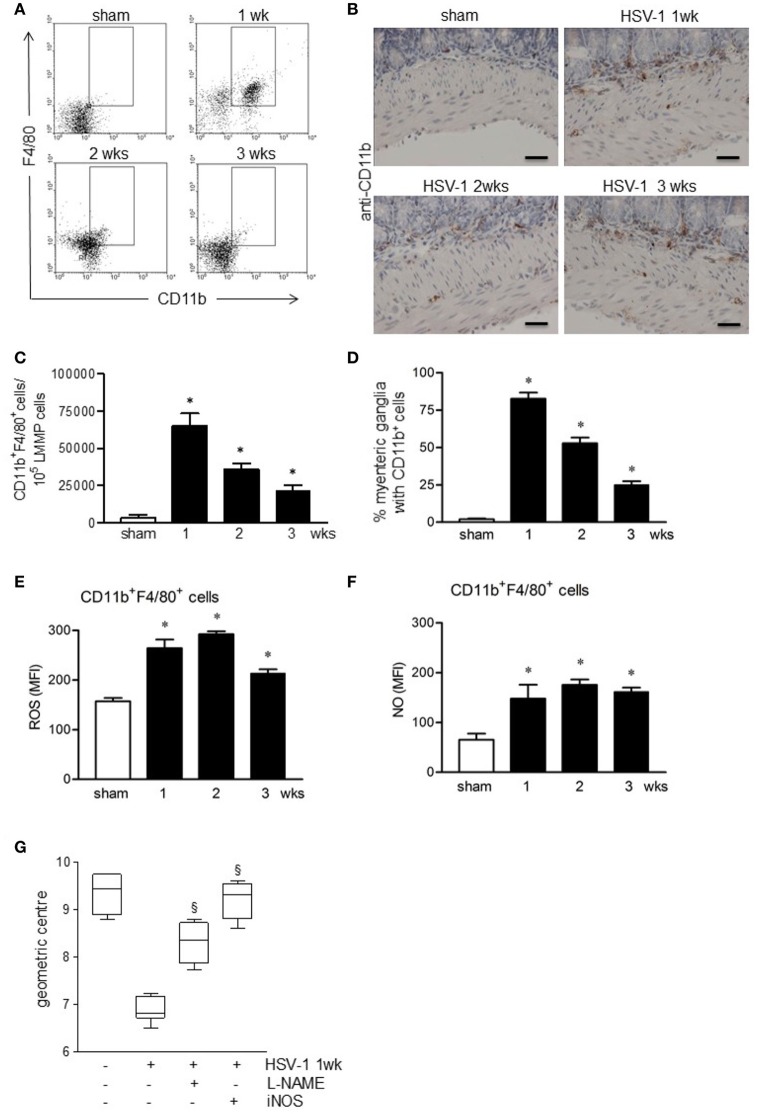
Activated macrophages infiltrate and damage the ENS of HSV-1 infected mice. **(A)** Freshly collected LMMP were digested and the resulting cell suspensions were labeled with anti-CD11b and anti-F4/80 antibodies and analyzed by flow cytometry. Representative images of five separate experiments. **(B)** Immunohistochemistry for CD11b on sections of ileum. Representative images of three separate experiments. Scale bars: 40 μm. **(C)** CD11b^+^F4/80^+^ cells analyzed by flow cytometry as in **(A)** were enumerated in 10^5^ collected events. **(D)** Myenteric plexuses reporting juxtaposed CD11b^+^ cells as in **(B)** were counted and normalized to the number of total myenteric plexuses. Data are reported as percentage. **(E)** Cells obtained from LMMP were incubated for 30 min at 37°C with H_2_DCFDA, a probe detecting intracellular ROS or **(F)** with DAF-FM to detect intracellular NO. Cells were then labeled with anti-CD11b and anti-F4/80 antibodies. Mean fluorescent intensity (MFI) was detected by flow cytometry in 10.000 events. *n* = 3–9 mice per group. ^*^Denotes *P* < 0.01 compared to sham infected mice. **(G)** Mice infected with HSV-1 strain 16 were intraperitoneally injected with L-NAME, a pan-NOS inhibitor, or with iNOS inhibitor (AR-C102222). At 1-week of infection, mice were administered with non-absorbable FITC-labeled dextran and sacrificed 60 min later. Intestinal transit was reported as the geometric center of distribution of the fluorescent probe throughout the ileum. Data are represented as mean ± SEM. *n* = 6 mice per group. ^§^Denotes *P* < 0.05 compared to 1-week HSV-1 infected mice.

We next investigated the role of the innate immunity in HSV-1 induced gastrointestinal dysmotility focusing on macrophages, cells controlling early phases of HSV-1 infection (Kodukula et al., 1999). Gut macrophages were studied by flow cytometry analysis on cells isolated from LMMP and characterized as CD11b^+^F4/80^+^ cells. As reported in Figures 6A–D, CD11b^+^F4/80^+^ cells were barely detectable in the LMMP of sham infected mice. Starting the first week following the IG inoculum of HSV-1 strain 16, significant increase in CD11b^+^F4/80^+^ macrophages was observed (Figures 6A,C). Immunohistochemistry on sections of the ileum revealed CD11b^+^ cells contiguous to the myenteric ganglia following IG viral inoculum (Figures 6B,D).

CD11b^+^F4/80^+^ macrophages infiltrating the LMMP of HSV-1 infected mice exhibited an activated phenotype since they generated free radicals, namely reactive oxygen species (ROS) and nitric oxide (NO; Figures 6E,F).

Taking into consideration also the data reported in Figure 3, to establish the role of macrophage-derived NO in functional alterations of the ENS, we administered HSV-1 infected mice with L-NAME, a non-selective NOS inhibitor. At 1-week post-HSV-1 infection, L-NAME administration ameliorated intestinal motility (Figure 6G). At the same, treatment with selective iNOS inhibitor, namely AR-C102222, significantly improved HSV-1 associated intestinal dysmotility (Figure 6G). No effects were observed in mice treated with vehicle alone (data not shown).

**Figure 7:**
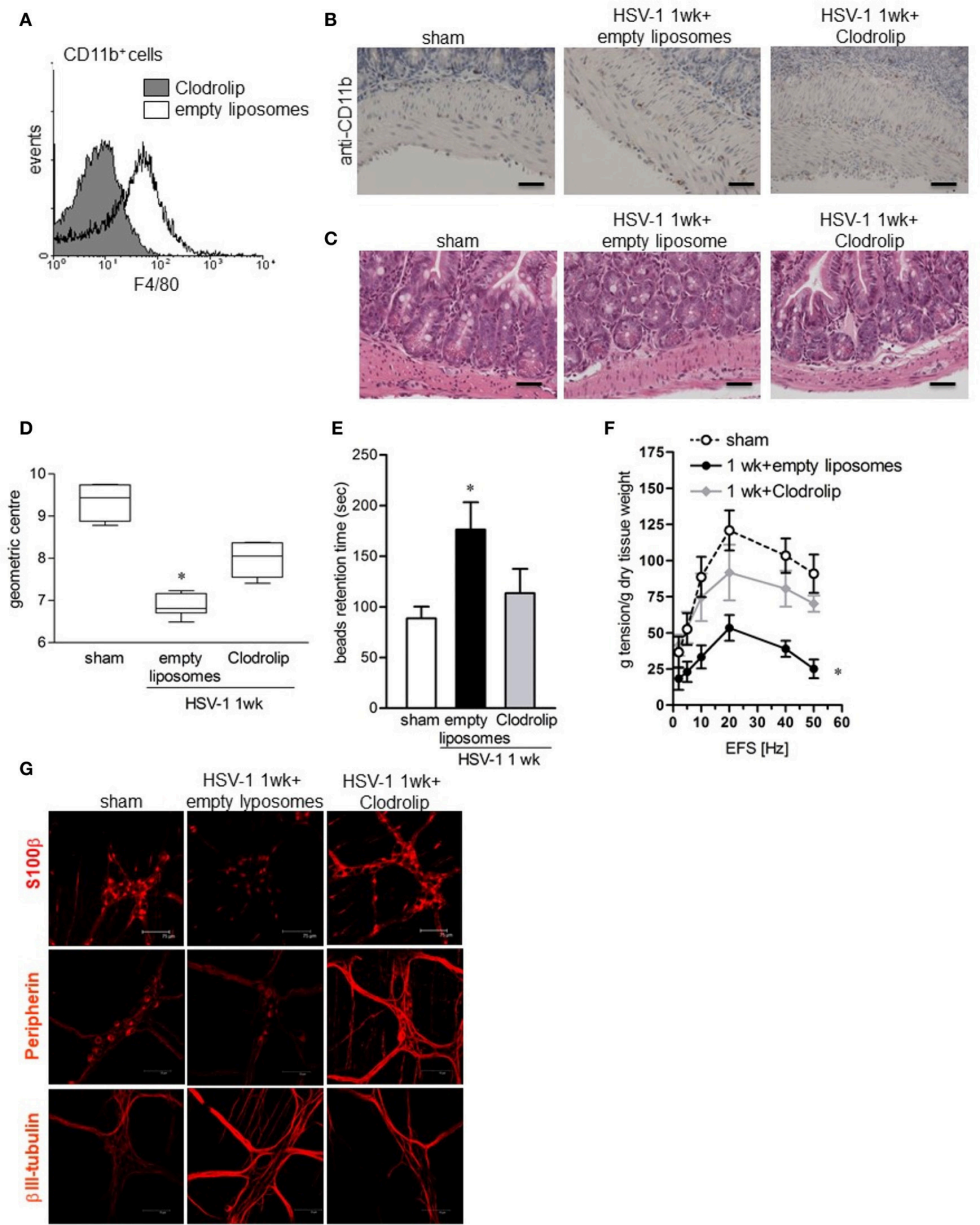
Depletion of macrophage in the LMMP ameliorates HSV-1-induced alterations of the ENS. **(A)** Cells were isolated form LMMP of 1-week HSV-1 infected mice treated with Clodrolip or empty liposomes as control. To confirm macrophages depletion, F4/80 expression was evaluated by flow cytometry in CD11b^+^ cells. **(B)** Sections of ileum obtained from 1-week HSV-1 infected mice treated with Clodrolip or empty liposomes were subjected to immunohistochemistry for CD11b. Scale bars: 40 μm. **(C)** Histological analysis of samples of ileum obtained from 1-week HSV-1 infected mice treated with empty liposomes or Clodrolip. Sections were subjected to H&E staining. Scale bars: 40 μm. Images are representative of three separate experiments. **(D)** At 1-week post-IG HSV-1 inoculum, mice injected with Clodrolip or empty liposomes were administered with non-absorbable FITC-labeled dextran and sacrificed 60 min later. Intestinal transit was reported as the geometric center of distribution of the fluorescent probe throughout the ileum. **(E)** Time (seconds, sec) required for expulsion of a glass bead inserted into the rectum in Clodrolip or empty liposomes treated mice at 1 week of IG infection. **(F)** EFS-elicited contractions in segments of ileum obtained from mice at 1 week of IG infection and treated with Clodrolip or empty liposomes. Data are reported as mean ± SEM. *n* = 6 mice per group. ^*^Denotes *P* < 0.01 compared to sham infected mice. **(G)** Immunohistochemistry for S-100β, peripherin, and βIII-tubulin on whole mount preparations of distal ileum obtained from 1-week HSV-1 infected mice and treated with Clodrolip or empty liposomes. Representative images of three separate experiments. Scale bars: 75 μm.

To ascertain the contribution of infiltrating macrophages on gastrointestinal neuromuscular dysfunction, circulating monocytes and tissue macrophages were depleted by intraperitoneal injection of clodronate-containing liposomes (Clodrolip) (Zeisberger et al., 2006). As shown in Figures 7A,B Clodrolip injection was effective at depleting CD11b^+^F4/80^+^ cells in LMMP when compared with empty liposome-injected mice. Macrophage depletion by Clodrolip did not alter histological appearance of intestine (Figure 7C) and almost completely restored gastrointestinal motility in HSV-1 infected mice in term of intestinal transit, colonic motility, and EFS-elicited contractions in ileum (Figure 7D–F). HSV-1 induced structural anomalies of the ENS ameliorated in Clodrolip-treated mice (Figure 7G).

### MCP-1 mediates macrophage recruitment in the LMMP of HSV-1 infected mice

**Figure 8:**
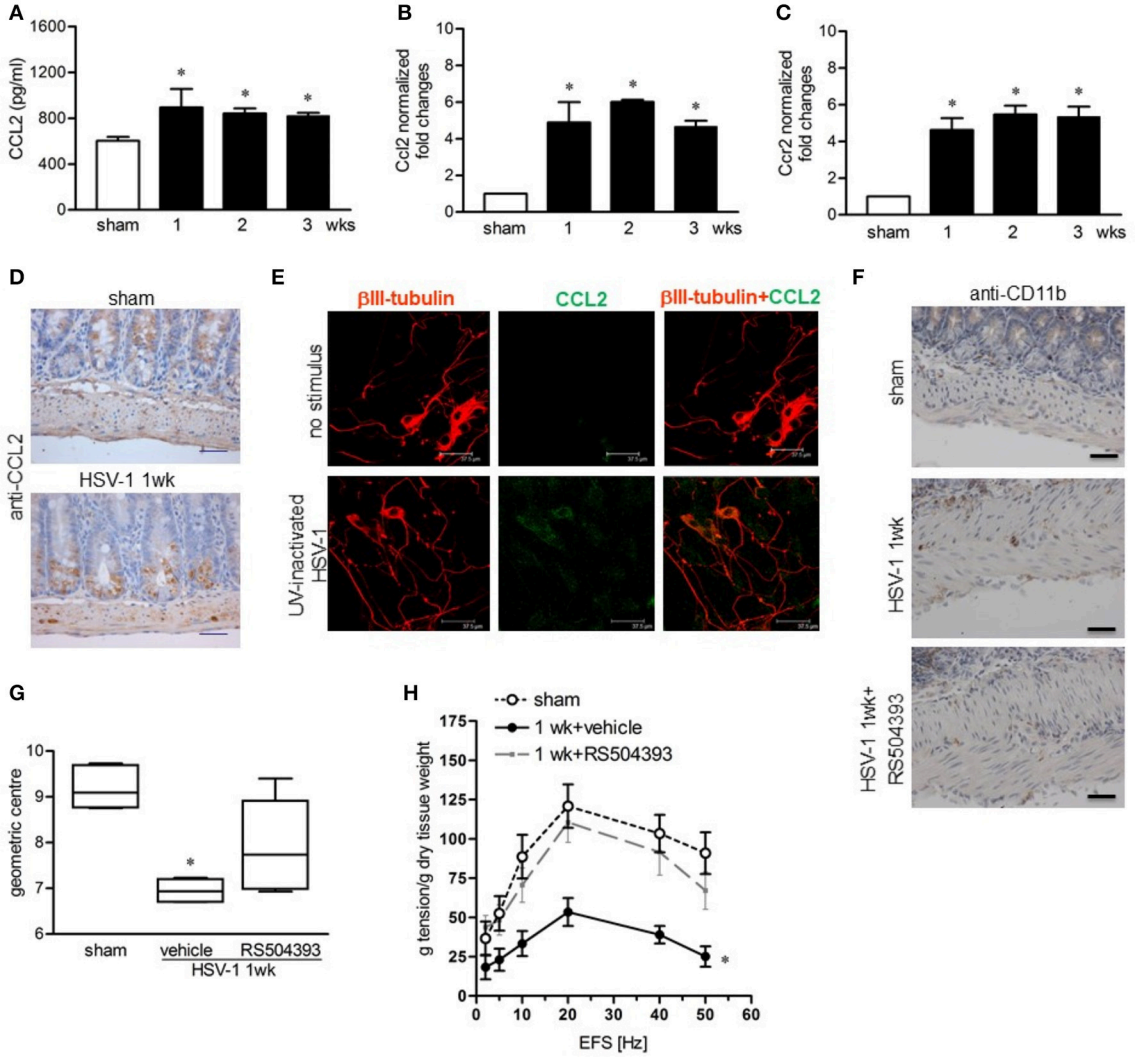
CCL2 is required for macrophage recruitment and intestinal dysmotility during HSV-1 infection **(A)** CCL2 levels were quantified by ELISA in LMMP extracts obtained from sham and HSV-1 strain 16 infected mice. **(B)** Quantitative RT-PCR analysis of *Ccl2* mRNA and **(C)** *Ccr2* mRNA in LMMP from sham and HSV-1 strain 16 infected mice. Data were normalized to ribosomal 18S RNA (*Rn18S*) and reported as fold change. **(D)** Immunohistochemistry for CCL2 on sections of ileum collected from sham and HSV-1 strain 16 infected mice. Scale bars: 40 μm. **(E)** Enteric neurons cultured from LMMP of non-infected mice were exposed for 16 h to UV-inactivated HSV-1. Cells were then fixed and probed with anti-βIII tubulin and anti-CCL2 antibodies. Cells were visualized under a confocal microscope. Scale bar = 37.5 μm. **(F)** At 1-week post-infection, mice were treated with RS504393, a highly selective CCR2 chemokine receptor antagonist. Immunohistochemistry on sections of ileum revealed decreased infiltration of CD11b^+^ cells. Scale bars: 40 μm. Images are representative of three separate experiments. **(G)** At 1-week post-IG HSV-1 inoculum, RS504393 or vehicle treated mice were administered with non-absorbable FITC-labeled dextran and sacrificed 60 min later. Intestinal transit was reported as the geometric center of distribution of the fluorescent probe throughout the ileum. **(H)** EFS-elicited contractions in segments of ileum of sham and HSV-1 infected mice treated with RS504393 or vehicle. Data are represented as mean ± SEM. *n* = 6–8 mice per group. ^*^Denotes *P* < 0.01 compared to sham infected mice.

Since monocyte chemoattractant protein-1 (MCP-1/CCL2) and its receptor CCR2 have been demonstrated to be involved in macrophage-dependent tissue damage (Gosling et al., 1999; Huang et al., 2001), we wondered whether the CCL2/CCR2 pathway was involved also in the HSV-1-induced damage of the ENS. CCL2 levels significantly increased in the LMMP following IG HSV-1 inoculum as determined by ELISA and quantitative RT-PCR (Figures 8A,B). In addition, Ccr2 mRNA levels were induced by HSV-1 infection (Figure 8C). Immunohistochemistry on sections of ileum revealed CCL2 positive cells located only in the mucosa of sham infected mice whereas 1 week following HSV-1 infection, CCL2 positive cells were detectable inside the myenteric ganglia (Figure 8D). In the attempt to identify the major source of CCL2 in the LMMP during HSV-1 infection, cultured enteric neurons were incubated with UV-inactivated HSV-1. Immunostaining demonstrated increased CCL2 in enteric neurons 16 h post-exposure to HSV-1 antigens (Figure 8E). To prove the functional role of the CCL2/CCR2 pathway on HSV-1 induced gastrointestinal dysmotility, HSV-1 infected mice were treated with RS504393, a highly selective CCR2 chemokine receptor antagonist (Kitagawa et al., 2004). Immunohistochemistry for CD11b^+^ cells in sections of ileum from HSV-1 infected mice treated with RS504393 proved a drastic reduction in macrophage recruitment (Figure 8F). Most interestingly, RS504393 treatment prevented the occurrence of HSV-1-induced gastrointestinal neuromuscular dysfunction (Figures 8G,H).

## Discussion

Functional gastrointestinal disorders are ascribed either directly or indirectly to altered activity of the ENS. Although degenerative neuropathy and intestinal dysmotility have been reported in patients suffering from irritable bowel syndrome (IBS) (Törnblom et al., 2002; Lindberg et al., 2009), the etiology and underlying pathophysiologic mechanisms remain largely unknown. Recently, Muller et al. found a tight communication between enteric neurons and resident gut macrophages under steady state conditions (Muller et al., 2014), arising the hypothesis that any inflammatory insult might perturb the function of neuronal network and vice versa neuronal damage might intensify gut inflammation. In the present study, we reported that following IG inoculum in mice, the neurotropic virus HSV-1 eludes the gastric barrier, reaches the gut lumen, and persists in enteric neurons (Figure 1). Through secretion of CCL2, infected neurons guide the recruitment and activation of macrophages which, via NO production, are responsible for the neuromuscular dysfunction (Figures 7, 8). Since HSV-1 DNA is intermittently shed in the saliva of even asymptomatic subjects and recovered in feces (Buddingh et al., 1953; Kaufman et al., 2005), these findings support the role of HSV-1 and viral-induced inflammation in gastrointestinal motor disorders.

Besides the classical antibacterial role, gut macrophages are devoted to maintaining the integrity of the enteric neurons, as also described in the central nervous system (Ginhoux et al., 2010; Cipriani et al., 2016). As opposed to the brain, macrophages at the interface between the host and the luminal environment are exposed to multiple factors and activate to clear dangerous agents or to recruit additional inflammatory cells (Phillips and Powley, 2012; Cipriani et al., 2016). Indeed, activated macrophages play an important part in the pathogenesis of intestinal dysfunctions both during experimentally induced inflammatory conditions (Galeazzi et al., 2001) and in presence of infectious agents (Kurt-Jones et al., 2004; Ellermann-Eriksen, 2005; Koyuncu et al., 2013). Inflammatory mediators such as reactive nitrogen and oxygen species (NO, ROS), interleukins and chemoattractant factors released by gut macrophages directly cause neuromuscular dysfunction in surgical manipulated ileum as well as in diabetic gastroparesis (Hurst and Collins, 1994; Khan and Collins, 1994; Wehner et al., 2007). In the present study we demonstated that the LMMP of mice at the early time of HSV-1 infection is infiltrated by activated macrophage, which generate ROS and NO (Figure 6). Depletion of CD11b^+^F4/80^+^ cells by Clodrolip as well as quencing the NO production by administration of NO synthase inhibitors significantly ameliorated neuromuscular dysfunction (Figures 6, 7) highlighting the key role of oxidative damage in enteric motorneuronal injuries. Indeed, NO directly or through peroxynitrite formation and tyrosine nitrosylation is involved in DNA damage and protein misfolding thus contributing to neurodegeneration and neuromuscular dysfunction (Nakamura et al., 2015). The ability of HSV-1 to establish a latent/abortive infection in enteric neurons, to induce an inflammatory response in the LMMP, and to alter the gastrointestinal motility has been previously reported in rats (Brun et al., 2010), thus confirming in two different animal models the involvement of HSV-1 in structural and functional alterations of peripheral nervous system. The amplitude and the functional consequences of HSV-1 infection of the ENS are however different in mice and rats probably because the differences in the subnetworks of chemokines and cytokines described in the two animal models (Du et al., 2017) that differently impact in the neuroplasticity of the ENS.

In the current study, we observed that following HSV-1 intragastrical inoculum the ENS underwent a variety of plastic changes (Figures 2, 3). Indeed, alterations of S100-β expression, type III intermediate filament peripherin, and the microtubule element βIII-tubulin (Figure 3B) have been described in several pathological conditions characterized by neuronal sufferance (Liem and Messing, 2009; Tischfield and Engle, 2010; Brun et al., 2013). Moreover, by Western blot analysis we reported increased expression of nNOS in myenteric plexus of HSV-1 infected mice associated with reduced intestinal transit and colonic motility (Figures 2B,C, 3C,D). The increased production of the inhibitory neurotransmitter NO occurs with concomitant reduction in the expression of ChAT at 1 week of infection (Figures 3C,D) when the nerve-mediated contractions (Figure 2D) and nerve coordinated gastrointestinal motility (Figures 2B,C) significantly differ from sham infected mice.

Like gut macrophages, enteric neurons synthesize and release cytokines, chemokines and growth factors to timely recruit and drive inflammatory cell adaptation to environmental perturbations (Coquenlorge et al., 2014; Burgueño et al., 2016; Gabanyi et al., 2016). During viral infection, neuronal-derived soluble factors take part in the development of neuroinflammation and fuel the inflammatory-immune circuits leading to tissue damage (Kumar et al., 2010; Chai et al., 2015). Similarly, besides the well-known type I and III interferon antiviral responses (Melchjorsen, 2012), neurons challenged with HSV-1 produce a specific panel of cytokines to ensure viral defense and regulate immune response (Hill et al., 2009; Gianni et al., 2013). In the present study, we show that enteric neurons exposed to HSV-1 secrete the chemokine CCL-2 to recruit and activate macrophages in the myenteric ganglia (Figures 7, 8) thus orchestrating the inflammatory response toward the pathogen. By promoting macrophage recruitment from the bloodstream to tissues, CCL2 plays a pivotal role in the pathogenesis of diseases characterized by mononuclear cells infiltration. For instance, Chen et al. reported a greater recruitment of inflammatory cells with significant larger areas of brain stroke during ischemic brain injury in mice over-expressing CCL2 as compared with wild-type controls (Chen et al., 2003). Accordingly, CCL2 deficiency reduces infiltration of macrophages into blood vessels, lung (Gosling et al., 1999; Dessing et al., 2007) and central nervous system in experimental animal models of inflammatory and neurodegenerative diseases (Huang et al., 2001; Muessel et al., 2002), including HSV-1 encephalitis (Kurt-Jones et al., 2004). In the gut, the activation of CCL2/CCR2 pathway has been described in several experimental colitis models (Yang et al., 2011) whereas CCL2 levels are increased in the colon of patients suffering from microscopic colitis and inflammatory bowel diseases (Arijs et al., 2011; Günaltay et al., 2015). Intriguingly, recent studies suggest that CCL2 contributes to the recruitment of specific macrophage subsets rather than *in situ* cell polarization thus shaping the extent of the inflammatory process (Takada et al., 2010).

Overall, in the present study we demonstrated that following an orogastric administration HSV-1 reaches and infects enteric neurons. HSV-1 infected neurons directly recruit inflammatory macrophages through the CCL2/CCR2 pathway that releasing ROS trigger changes in ENS neuroplasticity causing gastrointestinal dysmotility. Our data are in line with recent studies assigning to enteric neurons the modulation and timely adaptation of gut macrophages to environmental challenges (Muller et al., 2014; Gabanyi et al., 2016). Disruption in neuro-immune communication frequently results in gastrointestinal dysfunction as largely described in the intestinal mucosa of IBS patients where the count of infiltrating immune cells, including mast cells in close proximity to nerve fibers is coupled to the intensity of abdominal pain and gastrointestinal discomfort (Barbara et al., 2014). We can speculate that acute or chronic exposure of enteric neurons to neurotropic viruses, such as HSV-1, permanently disturbs the interplay between the ENS and the immune cells thus contributing, along with neuronal damage itself, to the pathogenesis of gastrointestinal diseases.

## Author contributions

All authors listed approved the manuscript for publication. PB and IC: designed research, performed research, analyzed data, and wrote the manuscript; MQ and AK: performed research; MG: analyzed data; PM, AP, VM, MS, GP, and AC critically read the manuscript; RS: provided Clodrolip and empty liposomes.

### Conflict of interest statement

The authors declare that the research was conducted in the absence of any commercial or financial relationships that could be construed as a potential conflict of interest.

## Abbreviations

LMMP: longitudinal muscle myenteric plexus
ENS: enteric nervous system
IN: intranasal
IG: intragastric
MCP-1/CCL2: monocyte chemoattractant protein-1
HSV-1: *Herpes simplex* virus type 1
WT: wild-type
L-NAME: Nω-Nitro-L-arginine methyl ester hydrochloride
iNOS: inducible nitric oxide synthase
EFS: electric field stimulation
NANCL: non-adrenergic non-cholinergic.
